# Pain, post-traumatic stress, and sleep disturbance in older adults with knee osteoarthritis: a path analysis of the mediating effect of depression and social support

**DOI:** 10.1186/s12877-026-07117-2

**Published:** 2026-02-13

**Authors:** Yabin Guo, Yanping Liu, Guojun Wang, Manman Su, Wenhui Chen, Yang Zhou

**Affiliations:** 1 Teaching and Research Section of Clinical Nursing, Xiangya Hospital of Central South University, Changsha, China; 2 Hunan Provincial Maternal and Child Health Care Hospital, Changsha, China; 3 Department of Operating Room, Xiangya Hospital of Central South University, Changsha, China; 4 Shenzhen Mental Health Center/Shenzhen Kangning Hospital, Shenzhen, China; 5 Department of Nursing, Xiangya Hospital of Central South University, Changsha, China; 6 Xiangya Nursing School of Central South University, Changsha, China

**Keywords:** Osteoarthritis, Knee, Sleep quality, Pain, Depression, Social Support, Stress Disorders, Post-Traumatic, Aged

## Abstract

**Background:**

Sleep disturbance is a prevalent and debilitating comorbidity in older patients with knee osteoarthritis (KOA), significantly impacting their quality of life. While pain is a known contributor, the complex interplay between physical symptoms, psychological distress, and social factors remains inadequately understood. This study aimed to determine the prevalence of poor sleep quality and to elucidate the intricate pathways through which pain, post-traumatic stress symptoms (PTSD), and social support influence sleep among community-dwelling older KOA patients.

**Methods:**

A multi-stage cluster random sampling method was employed to recruit 314 older patients with KOA from communities in Changsha. Data were collected on demographic characteristics, sleep quality (Pittsburgh Sleep Quality Index, PSQI), pain severity (Numerical Rating Scale, NRS), social support (Social Support Rating Scale), and symptoms of anxiety (Generalized Anxiety Disorder-7), depression (Patient Health Questionnaire-9), and PTSD (Posttraumatic Stress Disorder Checklist-Civilian Version, PCL-C). Poor sleep quality was defined as a PSQI global score > 7. Multivariable logistic regression and path analysis were performed to identify influencing factors and mediating pathways.

**Results:**

The prevalence of poor sleep quality (PSQI > 7) was 49.0% (154/314), with a mean cohort PSQI score of 8.18 ± 4.39. Bivariate analyses revealed that poor sleep quality was significantly associated with female gender, lower educational attainment, greater pain severity, higher levels of depressive and anxiety symptoms, higher PTSD scores, and lower social support (all *p* < .001). Path analysis, using a well-fitting model (TLI = 0.961, RMSEA = 0.070), identified depressive symptoms (total effect *β* = 0.396) and pain (total effect *β* = 0.329) as the most significant predictors of poorer sleep quality. The model further revealed that depressive symptoms played a crucial mediating role, channeling the indirect effects of both pain and PTSD onto sleep.

**Conclusion:**

Nearly half of the community-dwelling older adults with KOA suffer from poor sleep quality, underscoring a significant health burden. The risk is driven by a complex web of factors, not limited to pain. Our findings highlight that depressive symptoms and social support are critical mediators in the relationship between pain, trauma, and sleep. This suggests that effective management of sleep disturbances in this population necessitates a holistic, integrated approach that combines pain control with routine screening and management of psychological distress and reinforcement of social support systems.

## Introduction

In the context of global population aging, maintaining functional ability and well-being in later life is a public health priority [1] yet chronic conditions such as knee osteoarthritis(KOA) present a major barrier to achieving this goal. KOA represents a major public health challenge within aging populations worldwide [2, 3]. Affecting over 300 million people globally, KOA is a leading cause of chronic pain and functional disability, and it is projected to become a primary driver of global disability by 2030 [4]. Consistent with global trends, the burden is particularly acute in China as its population rapidly ages, with prevalence rates exceeding 12.7% [5]. The impact of KOA, however, extends far beyond joint pathology; it profoundly compromises mental health and overall quality of life, establishing it as a critical disabling condition [5].

Sleep disturbance represents a substantial comorbidity burden in patients with KOA. Evidence from the UK Biobank and Xiangya Osteoarthritis Cohort indicates a bidirectional relationship, where poor sleep quality—characterized by inadequate duration, insomnia, and daytime sleepiness—is associated with a 32% increased risk of developing KOA [6]. While the prevalence of poor sleep is approximately 35.9% in the general older population, it is disproportionately higher among those with KOA, affecting over 70% of patients [7, 8]. This high prevalence suggests a vicious cycle wherein chronic pain disrupts sleep architecture, while sleep deprivation conversely lowers pain thresholds and amplifies pain perception [9]. Furthermore, emerging evidence indicates that KOA-related sleep disturbances, similar to those in non-specific low back pain, are not solely attributable to nociceptive input. Instead, they likely stem from a multifactorial etiology within a complex bio-psycho-social framework involving intricate interactions between physical symptoms, psychological distress, and social factors [10, 11].

The relationship between pain and sleep is unlikely to be purely direct; rather, it is modulated by a complex interplay of psychosocial factors [12, 13]. A growing body of evidence has linked poor sleep quality in KOA patients to psychological distress [14], such as depression [15] and anxiety [16], and to psychosocial resources, like social support [4].Moreover, post-traumatic stress disorder (PTSD) represents a significant, yet under-explored, psychological determinant of sleep disturbance in older adults with KOA [17]. Despite the established comorbidity between chronic musculoskeletal pain and PTSD, their specific interaction within this geriatric population remains ill-defined. Evidence indicates that PTSD is strongly associated with insomnia and fragmented sleep [18]., likely driven by shared mechanisms such as physiological hyperarousal, heightened threat appraisal, and avoidance behaviors. In older adults, traumatic events—ranging from falls and surgeries to traffic accidents—can precipitate or exacerbate both KOA and PTSD. This complex relationship is elucidated by the mutual maintenance model [19], which posits that chronic pain acts as a persistent somatic reminder of trauma. This reminder perpetuates hyperarousal and intrusive symptoms, thereby severely compromising sleep architecture [20]. Therefore, chronic pain itself acts as a stressor that may trigger or perpetuate PTSD, thereby further exacerbating sleep difficulties.

Despite these established associations, the underlying mechanisms linking pain to sleep disturbance in the KOA population remain insufficiently characterized. Elucidating these pathways is clinically imperative, as it facilitates a paradigm shift from exclusively pain-centric management to holistic, mechanism-based interventions. Specifically, identifying modifiable mediators could inform targeted strategies—ranging from psychological therapies to social support enhancement—thereby optimizing sleep and well-being in this vulnerable cohort.

Consequently, this cross-sectional study aimed to: (1) assess the prevalence of sleep disturbance among community-dwelling older adults with KOA in Changsha, China; and (2) employ path analysis to evaluate the mediating effects of depressive symptoms and social support on the relationships between pain intensity, PTSD, and sleep quality. We hypothesized that higher pain intensity and PTSD would be directly associated with poorer sleep quality, while depressive symptoms and social support would serve as significant mediators in these pathways.

## Materials and methods

### Study design and participants and eligibility criteria

This cross-sectional study was conducted in Changsha, the capital city of Hunan Province, China, from October to November 2021. The sample only included older patients with KOA aged 65 and above in the urban community of Changsha city. The inclusion criteria were as follows: (1) age ≥ 65, (2) consistent with the diagnostic criteria for KOA in Guidelines for the diagnosis and treatment of osteoarthritis in China (2018 edition) [21], (3) able to participate in the study. The Exclusion criteria were (1) presence of other severe musculoskeletal disorders (e.g., rheumatoid arthritis, hip osteoarthritis), (2) history of severe knee trauma or surgery, (3)diagnosed with severe cognitive impairment (e.g., dementia) or psychiatric disorders that would preclude accurate completion of questionnaires, (4) suffering from terminal illnesses.

### Sampling procedure and participant recruitment

**Fig. 1:**
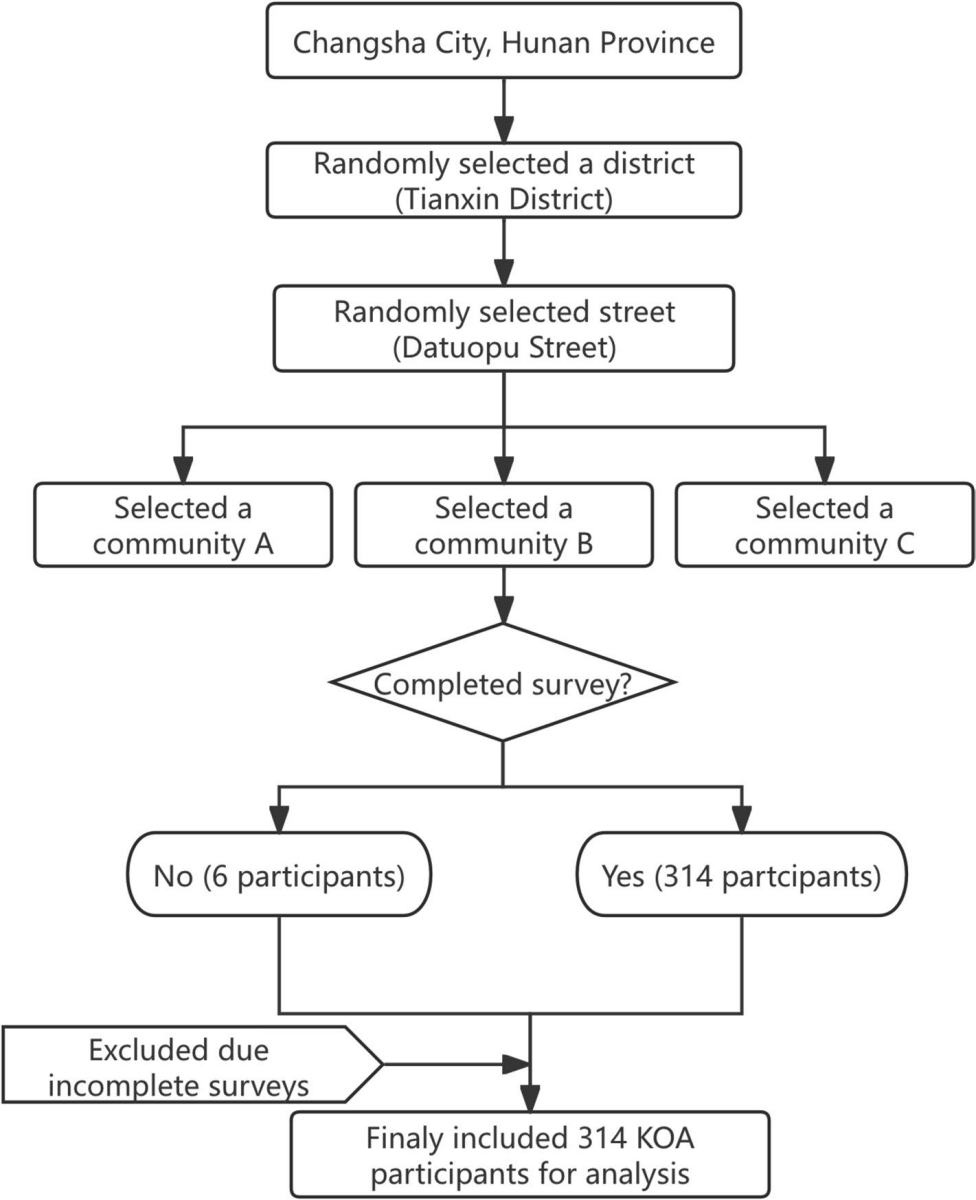
Illustrates the recruitment process of the participants

A multi-stage cluster random sampling strategy was employed to recruit a representative sample of urban older KOA patients. The procedure was conducted in four stages (Fig. 1):

Stage 1: District Selection. From a complete list of the five administrative districts in Changsha, one district was selected using a computer-generated random number sequence.

Stage 2: Street Selection. Within the selected district, a comprehensive list of all administrative streets was obtained. One street was then randomly chosen from this list using the same computer-generated randomization method.

Stage 3: Community Selection. Subsequently, from a list of all urban communities located on the selected street, three communities were randomly selected.

Stage 4: Participant Recruitment. In the three selected communities, we collaborated with the local community health service centers. First, we obtained a list of all registered residents aged 65 and above who had a recorded diagnosis of KOA. This formed our sampling frame of potential participants. Then, trained research assistants contacted these individuals by telephone to explain the study's purpose and procedures and to screen them for eligibility based on the inclusion and exclusion criteria. All eligible and willing individuals were invited to the community health center to complete the questionnaires.

### Sample size determination

The required sample size was calculated using the formula for a single proportion: n = (Zα/2)^2^ * p * (1-p)/d^2^. Based on a previous study reporting a 39.3% prevalence (p) of sleep disorders among KOA patients [22], and setting the confidence level at 95% (Zα/2 = 1.96) and the absolute precision (d) at 0.059, the minimum required sample size was calculated to be 263.

To account for potential non-responses or incomplete data, we anticipated a 15% dropout rate. Therefore, the target sample size was adjusted to 310 [calculated as 263/(1—0.15)].

### Measures

#### Demographic characteristics

We used a self-designed questionnaire to collect the participants’ demographic characteristics, including gender, age, education, marital status, number of children, employment, family annual income, religious belief, smoking, diagnosis of KOA, and four related symptoms of KOA (recurrent knee pain in the past month; knee joint stiffness ≤ 30 min in the morning; a fricative sound (feeling) exists in the knee joint when moving; radiographs (standing or weight-bearing) show narrowing of the joint space, subchondral sclerosis and/or cystic degeneration, and osteophyte formation at the joint margin).

#### Pittsburgh sleep quality index

Sleep quality in the past month before the investigation was measured by the Pittsburgh Sleep Quality Index (PSQI). This instrument is frequently used to screen sleep quality, with scores ranging from 0 to 21. A higher score indicates more severe symptoms and poor sleep quality. The PSQI has been used in a previous study in China and exhibited satisfactory reliability and validity, the test–retest reliability coefficient was 0.99, the split-half reliability coefficient was 0.82 and the Cronbach's α coefficient was 0.85 [23]. Poor sleep quality was defined as a PSQI global score > 7.

#### Numerical rating scale

We used the Numerical Rating Scale (NRS) to access the participants’ level of knee pain. The scale comprises 11 numbers from 0 to 10, describes the intensity of the pain, and the higher the number, the more severe the pain. A score of 0 indicates no pain, 1 to 3 are classified as mild pain (pain does not affect sleep), 4 to 6 were classified as moderate pain, 7 to 10 were classified as severe pain [24].

#### Social support rating scale

The level of social support was measured by the 10-item Social Support Rating Scale (SSRS). The scale was reflected in three dimensions; namely, objective support (3 items), subjective support (4 items) and the utilisation of social support (3 items). The total scores ranging from 17 to 74, wherein a higher score indicates further social support the participants have received. Liu Jiwen et al. [25] showed that the correlation coefficient between total scale and three subscales were 0.724–0.835, and the Cronbach's α coefficients of total and subscales were 0.896, 0.849, 0.825, 0.833, respectively.

#### Generalized anxiety disorder-7

Anxiety symptoms of the participants in the last two weeks were measured by Generalized Anxiety Disorder 7-item (GAD-7) scale. The scale comprises seven items, response options for each item range from 0 to 3 on a four-point Likert scale (0 = not at all, 1 = several days, 2 = more than half the days and 3 = nearly every day). The total scores ranging from 0 to 21. This instrument showed satisfactory reliability and validity in a previous study in China [26], the internal consistency coefficient was 0.92, the test–retest reliability coefficient was 0.83。

#### Patient health questionnaire-9

The participants’ depressive symptoms in the last two weeks were measured by the Patient Health Questionnaire-9 (PHQ-9). The scale comprises nine items, respondents indicated, on a 0–3 scale, the frequency with which they experienced the following nine symptoms: anhedonia, depressed mood, sleep disturbance, fatigue, appetite changes, low self-esteem, concentration problems, psychomotor disturbances, and suicidal ideation. The total scores ranged from 0 to 27. This instrument showed satisfactory reliability and validity in a previous study in China, the Cronbach's α coefficient was 0.84 [27].

#### Post-traumatic stress disorder checklist-civilian version

Post-traumatic experiences of the participants in daily lives were screened by the Posttraumatic Stress Disorder Checklist-Civilian Version (PCL-C). The scale consists of 17 items, response options for each item range from 1 to 5 on a five-point Likert scale. The total scores ranged from 17 to 85, with a higher score indicating more severe PTSD symptoms. This instrument showed satisfactory reliability and validity in a previous study in China, the Cronbach's α coefficient was 0.94 [28], the test–retest reliability coefficient was 0.80.

### Procedures and quality control

Face-to-face interviews was conducted, and WeChat survey to collect information were held from 1 October 2021 to 30 November 2021. Before the WeChat survey, the participant was informed about the background and purpose of our research and signed an informed consent form detailing their rights. The questionnaire was modified and optimised through professional consultation and preliminary investigation to ensure the scientific, comprehensive and feasible nature of the questionnaire. All items of the questionnaire were set as mandatory items, and the order of items was sorted as logically as possible, which was convenient for the subjects to fill in.

### Statistical methods

Statistical analyses were conducted using SPSS 22.0 (SPSS/IBM, Chicago, IL). The demographic characteristics was compared between 154 sleep disorder and 160 no sleep disorder among older patients with KOA participants using the t test, χ2 test or Wilcoxon rank-sum test. Risk factors for sleep disorders were explored using binary logistic regression. The significance level was set at *p* < 0.05. The inclusion and exclusion criteria were 0.05 and 0.10, respectively.

Path analysis was conducted using AMOS 24.0 to examine the hypothesis that the relationship between pain, PTSD, social support, depressive symptoms, anxiety symptoms, and sleep. Pain, PTSD symptoms, social support, depressive symptoms, anxiety symptoms and sleep quality were included in the models. The minimum fit function χ2 value (CMIN), CMIN/DF, comparative fit index (CFI), incremental fit index (IFI) and root mean square error of approximation (RMSEA) with 90% confidence intervals were used to estimate the model fit.

## Results

### Demographic characteristics, anxiety, social support, depression, hopelessness and ptsd in sleep disorder and no sleep disorder

A total of 314 community-dwelling older patients with KOA were included in the analysis. The mean global PSQI score for the entire cohort was 8.18 ± 4.39. Based on the established cutoff (PSQI > 7), the prevalence of poor sleep quality was 49.0% (*n* = 154). As shown in Table 1, baseline characteristics were compared between participants with poor sleep quality (*n* = 154) and normal (*n* = 160). The two groups did not differ significantly in age, marital status, number of children, employment status, family annual income, religious belief, smoking history(all *p* > 0.05). However, the poor sleep quality group contained a significantly higher proportion of females (e.g., 70.1% vs. 51.9%, *p* = 0.001) and individuals with a primary school education or less (e.g., 58.4% vs. 39.4%, *p* = 0.001). Furthermore, patients in the poor sleep quality group exhibited a significantly greater burden of clinical symptoms. A significant dose–response relationship was found between pain severity and the prevalence of poor sleep quality (χ^2^ = 25.805, *p* < 0.001). A similarly strong association was evident for mental health indicators. As anxiety levels increased, the prevalence of poor sleep rose significantly from 39.0% in the no-anxiety group to 75.0% in the moderate-to-severe anxiety group (χ^2^ = 19.530, *p* < 0.001). The impact of depressive symptoms was particularly striking (χ^2^ = 30.868, *p* < 0.001): while the prevalence of poor sleep was 36.0% among patients with no depression, it surged to 80.0% among those with moderate-to-severe depression. Concurrently, their mean score for PCL-C was significantly higher (30.73 ± 11.31) than that of the good sleep quality group (26.20 ± 10.60; *t* = −4.084, *p* < 0.001).

**Table 1:** Demographic characteristics, Anxiety, social support, depression, hopelessness and PTSD between 154 sleep disorder and 160 no sleep disorder among elderly patients with knee osteoarthritis

Demographic characteristics	Sleep disorder	No sleep disorder	χ^2^/Z/t	*P*
	(*n* = 154)	(*n* = 160)		
Gender, n(%)			10.975	0.001^**^
Female	108(56.5%)	83(43.5%)		
Male	46(37.4%)	77(62.6%)		
Age(years), n(%)				
65–74	93(44.7%)	115(55.3%)	4.630	0.099
75–84	50(57.5%)	37(42.5%)		
≥ 85	11(57.9%)	8(42.1%)		
Education, n(%)			15.622	0.001^**^
Primary school and below	90(58.8%)	63(41.2%)		
junior high school	27(32.5%)	56(67.5%)		
technical secondary school/high school	24(44.4%)	30(55.6%)		
Senior college and above	13(54.2%)	11(45.8%)		
Marital status, n (%)			2.512	0.072
Currently married	103(46.2%)	120(53.8%)		
Not currently married	51(56.0%)	40(44.0%)		
Number of children, n (%)			2.765	0.251
1 and below	29(45.3%)	35(54.7%)		
2	52(44.8%)	64(55.2%)		
3 and above	73(54.5%)	61(45.5%)		
Employment, n (%)			0.246	0.884
Brain worker	40(47.1%)	45(52.9%)		
Manual laborer	82(50.3%)	81(49.7%)		
Unemployed	32(48.5%)	34(51.5%)		
Family annual income (RMB), n (%)			6.175	0.103
< 1000	51(60.0%)	34(40.0%)		
1000–3000	57(44.5%)	71(55.5%)		
3001–5000	33(43.4%)	43(56.6%)		
> 5000	13(52.0%)	12(48.0%)		
Religious belief, n (%)			2.857	0.067
Yes	18(64.3%)	10(35.7%)		
No	136(47.6%)	150(52.4%)		
Smoking, n (%)			1.003	0.192
Yes	33(44.0%)	42(56.0%)		
No	121(50.6%)	118(49.4%)		
Pain level, n (%)			25.805	0.000^**^
Mild pain	74(38.5%)	118(61.5%)		
Moderate pain	57(60.6%)	37(39.4%)		
Severe pain	23(82.1%)	5(17.9%)		
Anxiety level, n (%)			19.530	0.000^**^
No anxiety	71(39.0%)	111(61.0%)		
Mild anxiety	62(59.6%)	42(40.4%)		
Moderate and severe anxiety	21(75.0%)	7(25.0%)		
Depression level, n (%)			30.868	0.000^**^
No depression	62(36.0%)	110(64.0%)		
Mild depression	60(58.8%)	42(41.2%)		
Moderate and severe depression	32(80.0%)	8(20.0%)		
Social support, mean ± SD	38.10 ± 8.05	40.86 ± 9.35	2.622	0.009^**^
PCL-C, mean ± SD	30.73 ± 11.31	26.20 ± 10.60	−4.084	0.000^**^

^*^P < 0.05, ^**^P < 0.01

### Risks of sleep disorders among older KOA patients: multivariable regression

Binary logistic regression shows that the independent variables of sleep disorder in this model were as follows: gender, education, pain level (mild pain, moderate pain, severe pain), anxiety level (no anxiety, mild anxiety, moderate and severe anxiety), depression level (no depression, mild depression, moderate and severe depression), PTSD, social support, (the PTSD and social support scores were used as continuous variables). The three variables in the final model were as follows: gender (*OR* = 2.00, 95% *CI* = [1.20, 3.32]), moderate pain (*OR* = 1.93, 95% *CI* = [1.13, 3.31]), severe pain (*OR* = 6.08, 95% *CI* = [2.13, 17.40]), mild depression (*OR* = 2.27, 95% *CI* = [1.34, 3.86]) and moderate and severe depression (*OR* = 6.68, 95% *CI* = [2.82, 15.87]) (Table 2).

**Table 2:** Risks of sleep disorders among elderly patients with knee osteoarthritis

Independent variables	*P*	OR[95%CI]
Gender		
Male		1
Female	0.007	2.00[1.20, 3.32]
Pain level		
Mild pain		1
Moderate pain	0.017	1.93 [1.13, 3.31]
Severe pain	0.001	6.08[2.13, 17.40]
Depression		1
No depression		
Mild depression	0.002	2.27 [1.34, 3.86]
Moderate and severe depression	0.000	6.68 [2.82, 15.87]

### Testing the structural equation model

Based on the multivariable regressive model results, we established two path analysis models to explore the relationships between pain, PTSD, social support, depressive symptoms, anxiety symptoms, and sleep. The theoretical model was not well-validated (Model 1). The χ2 test exhibited a significant probability of > 0.05, χ2(1) = 3.211, *p* = 0.012. The GFI analysis showed that the model fit was high: GFI = 0.987, AGFI = 0.930, TLI = 0.944 and RMSEA = 0.084. The PTSD had no direct effects on sleep and social support, anxiety symptoms had no direct effect on sleep.

**Fig. 2:**
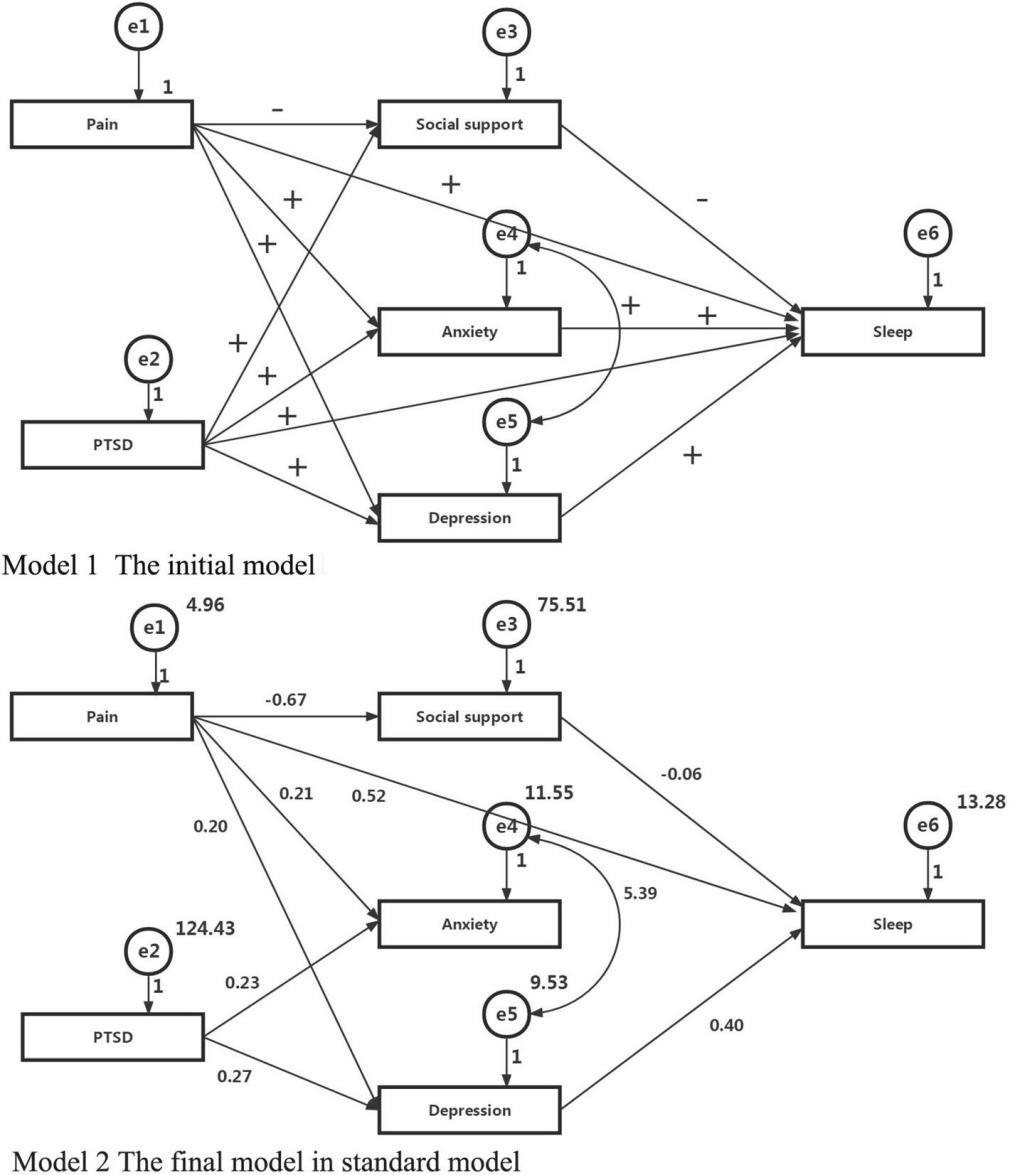
The structural equation model of this study. Model 1: Pain not only has a direct effect on suicide but also have an indirect effect by increasing the risk of poor sleep. PTSD, social support, depression and anxiety mediate the relationship between pain and sleep. The mediating effects of these factors on the relationship between pain and sleep were examined using a structural equation model: CMIN = 12.843, CMIN/DF = 3.211, p = 0.012, GFI = 0.987, AGFI = 0.930, TLI = 0.944 and RMSEA = 0.084. Model 2: The relationship between Pain and sleep is mediated by PTSD, social support, anxiety symptoms, and depressive symptoms. The mediating effects of these factors on the relationship between Pain and sleep were examined using a structural equation model: CMIN = 15.138, CMIN/DF = 2.523, p = 0.019, GFI = 0.984, AGFI = 0.945, TLI = 0.961 and RMSEA = 0.070

We removed the paths with no direct effect, and created Model 2. The verification analysis of the structural equation model proved that the modified model was well validated (Model 2). The χ2 test showed a significant probability of > 0.05, χ2(1) = 2.523, *p* = 0.019. The GFI analysis showed that the model fit was high: GFI = 0.984, AGFI = 0.945, TLI = 0.961 and RMSEA = 0.070. The standardised total effects of the predictors on sleep were as follows: pain = 0.329, PTSD = 0.107, social support = −0.055, depressive symptoms = 0.396 (see Fig. 2). Pain and depression had a direct positive effect on sleep, whilst social support had a direct negative effect on sleep. Pain can indirectly affect sleep through social support, anxiety and depression, in which social support, anxiety and depression play a chain-mediating role in the relationship between pain and sleep. Post-traumatic stress disorder can indirectly affect sleep through depression, in which depression plays a chain mediating role in the relationship between post-traumatic stress disorder and sleep. Pain and post-traumatic stress disorder had a direct positive effect on anxiety, but anxiety had no direct effect on sleep.

## Discussion

The primary objective of this study was to determine the prevalence of sleep disturbance in community-dwelling older adults with KOA while delineating the complex interplay of contributing factors. In interpreting these findings, the discussion categorizes risk factors into non-modifiable and modifiable domains to better inform targeted clinical interventions.

### The current situation of sleep disorders in older patients with KOA was unoptimistic

The prevalence of sleep disorders was 49.0% amongst community older patients with KOA. The prevalence observed in the present study surpasses findings from a previous domestic study involving 102 preoperative patients with KOA, which reported a rate of 39.3% [22]. This discrepancy may be attributed to the multifaceted burden faced by community-dwelling older adults, characterized by persistent pain, functional limitations, and the progressive nature of the disease. Furthermore, diminished self-efficacy and self-worth may exacerbate these issues. Ultimately, older adults with KOA endure sustained stress across physical, psychological, economic, and social domains, collectively contributing to poorer sleep outcomes. The reason may be that patients with KOA suffer from knee pain and dysfunction, repeated illness, poor prognosis, self-care ability and sense of self-worth decreased. KOA patients suffer from physical, psychological, economic, and social stress for a long time. KOA patients have a low level of self-efficacy [29] and lack of social support [30], thus exhibiting a low level of mental resilience, accompanied by negative emotions. These negative stressors affect the ability and confidence of patients to adapt to the disease, and aggravate the occurrence of sleep disorders.

### Demographic characteristics and risk stratification: focus on women and vulnerable groups

In this study, the prevalence of sleep disturbances was significantly higher in female participants compared to males (60.8% vs. 39.2%). Binary logistic regression confirmed female gender as an independent risk factor for sleep disorders (*OR* = 2.00), a finding consistent with previous research indicating that older women are disproportionately affected by poor sleep [31]. This disparity may be attributed to a confluence of biopsychosocial factors. Compared to their male counterparts, older females often report higher rates of depressive symptoms and negative life events while receiving less social support. Furthermore, biological mechanisms such as hormonal fluctuations, combined with heavier caregiving burdens and distinct help-seeking behaviors, may heighten their vulnerability to emotional distress and sleep disruption. Consequently, targeted interventions for sleep health in older women with KOA are warranted. Notably, age itself was not a significant predictor of sleep disturbance in our univariate analysis (*p* > 0.05). This suggests that sleep impairments in this population are driven primarily by disease-specific burdens—such as pain and psychological distress—rather than being an inevitable consequence of the aging process.

### Pathways of action for modifiable factors: the interactive effects of pain, ptsd, depression and social support

In contrast to immutable demographic characteristics, factors such as pain, depression, PTSD, anxiety, and social support are modifiable and amenable to intervention. The structural equation model constructed in this study elucidates the complex interplay among these variables, thereby identifying precise targets for clinical management and therapeutic strategies.

#### Direct or indirect effect of pain on sleep quality among community older patients with KOA

Our data isolate pain as the dominant, modifiable driver of poor sleep. Patients reporting severe pain faced a six-fold increase in sleep disturbance risk (*OR* = 6.08) compared to those with mild symptoms. While this echoes findings from Nigerian cohorts [32], the effect size we observed is considerably more pronounced. Path analysis indicates that pain disrupts sleep partly through psychological mediators, yet its direct impact is undeniable. We suspect this reflects central sensitization, where chronic nociceptive input triggers neuroplastic changes that amplify pain perception [33]. This state of physiological hyperarousal often involves abnormal activity in brain regions regulating both pain and sleep—such as the thalamus and limbic system [34]—thereby directly fragmenting sleep. Clinically, this implies that managing sleep in older adults with KOA requires a dual approach: addressing nociception while concurrently targeting emotional distress and psychosocial deficits.

 Furthermore, the patients in our study with severe sleep disturbances may overlap substantially with the 'refractory' population described by Conrozier et al. [35] who respond poorly to standard therapies like hyaluronic acid. Consequently, when evaluating novel analgesic interventions for refractory cases—such as the carboxymethyl chitosan therapies noted by Manocchio et al. [36]—we recommend incorporating sleep quality as a primary secondary outcome to comprehensively assess therapeutic efficacy.

#### The mediating and amplifying effects of psychological factors

This study confirms that depression and PTSD serve as key mediating variables linking pain to sleep disorders. This study found that depression and PTSD maybe an important mediating variables linking pain to sleep disturbances. The prevalence of depression in this study was 45.2%. This finding is higher compared with previous studies in Australia. In a study of 397 participants with symptomatic KOA reporting 25.4% participants had depression [37]. These differences in results could be due to more physical illness, COVID-19 pandemic environment, or age. The binary logistic regression shows that the more severe the level of depression, the higher the risk of developing sleep disorders, mild depression (compared with no depression, *OR* = 2.27), moderate and severe depression (compared with no depression, *OR* = 6.68) were independent risks of sleep disorder among older patients with KOA. This result is consistent with previous studies on patients with KOA in Nigeria, reporting depression is a risk factor for sleep disorders in patients with KOA (*OR* = 1.11) [32]. The structural equation model showed that depression directly elevated poor sleep risk older patients with KOA, which explain our results that depression was associated with poor sleep directly after controlling for these mediating factors with a binary logistic regression. Our path analysis suggests that depressive symptoms serve as a key mediating variable linking pain, trauma symptoms, and sleep quality. Whilst this cross-sectional study cannot establish causality, the biopsychosocial model posits that persistent pain depletes psychological resources, inducing depressive mood, which may subsequently impair sleep by disrupting the regulatory function of the hypothalamic–pituitary–adrenal axis. This suggests that depressive symptoms may act as an “amplifier” in the deterioration of sleep caused by pain.

#### Mediating role of anxiety and post-traumatic stress disorder on sleep quality

In this study, we examined PTSD not as a combat-related phenomenon, but as a consequence of chronic illness in older adults [38]. Consistent with the Mutual Maintenance Model [19], chronic pain itself functions as a persistent physical stressor. While univariate analysis indicated elevated anxiety and PTSD levels in patients with sleep disorders, binary logistic regression did not identify these as independent risk factors. Structural equation modeling resolved this discrepancy, revealing that PTSD exerts a fully mediated effect on sleep via depressive symptoms rather than a direct impact. This suggests that in older adults with KOA, pain-induced hyperarousal—such as startle responses and intrusive thoughts—precipitates emotional dysregulation, which subsequently degrades sleep quality. Consequently, clinical interventions must address anxiety and PTSD as critical upstream factors; early management to prevent their progression into depression is essential for improving sleep outcomes.

### Implications

Our results have important implications in sleep problem prevention amongst older KOA patients. Just as the management of NSLBP has shifted from a purely biomedical model towards a bio-psycho-social model, our findings underscore that the management of sleep disturbances in KOA should likewise adopt a multi-dimensional approach. To confirm our results in clinical practice, interventions should target two primary modifiable factors. First, priority must be given to the diminution of pain through pharmacological or physical therapies, addressing the primary physical disruptor of sleep. Second, alongside screening for depression, clinicians should focus on the direct amelioration of sleep disorders via evidence-based protocols like Cognitive Behavioral Therapy for Insomnia. By integrating these treatments with enhanced social support, we can effectively interrupt the pain-insomnia vicious cycle and validate the causal pathways identified in this study.

### Limitation of the study

However, two limitations should be considered when interpreting our results. Firstly, the sample in this study comprises older adults (60 or above) living in three urban communities in Hunan, China. It is not a nationally representative sample of the urban older in China and does not provide information on older rural adults. Risk factors of sleep disorder may differ in different parts of China. Secondly, this is a cross-sectional study. Our results only indicate the correlations amongst pain and PTSD, social support, anxiety symptoms, depressive symptoms and sleep disorder. Further intervention studies aimed at preventing or treating sleep disorder in this population through providing social connections and treating any pain, depression, anxiety, PTSD are warranted. Finally, this study employed solely subjective questionnaires to assess sleep quality. Whilst the PSQI is a validated instrument, subjective reporting may not fully capture objective sleep physiological parameters. Future research incorporating objective measurement techniques—such as polysomnography or actigraphy—would facilitate deeper analysis of the specific sleep phase and structural abnormalities observed in patients with KOA.

## Conclusion

Pain can elevate the risk of sleep disorder by increasing depressive symptoms, decreasing social support; PTSD can elevate the risk of sleep disorder by increasing depressive symptoms; anxiety and depression are strongly correlated. Efforts for sleep disorder prevention should be integrated with strategies to treat any pain, depression, anxiety and PTSD, along with psychological interventions.

## Data Availability

The data that support the findings of this study are available from the corresponding author upon reasonable request.
